# Dimethyl Sulfoxide (DMSO) Exacerbates Cisplatin-induced Sensory Hair Cell Death in Zebrafish (*Danio rerio*)

**DOI:** 10.1371/journal.pone.0055359

**Published:** 2013-02-01

**Authors:** Phillip M. Uribe, Melissa A. Mueller, Julia S. Gleichman, Matthew D. Kramer, Qi Wang, Martha Sibrian-Vazquez, Robert M. Strongin, Peter S. Steyger, Douglas A. Cotanche, Jonathan I. Matsui

**Affiliations:** 1 Department of Neuroscience, Pomona College, Claremont, California, United States of America; 2 Department of Molecular and Cellular Biology, Harvard University, Cambridge, Massachusetts, United States of America; 3 Oregon Hearing Research Center, Department of Otolaryngology, Oregon Health & Science University, Portland, Oregon, United States of America; 4 Department of Chemistry, Portland State University, Portland, Oregon, United States of America; 5 Harvard-MIT Health Sciences and Technology, Cambridge, Massachusetts, United States of America; 6 Harvard Noise-Induced Hearing Loss Research Group, Harvard School of Public Health, Boston, Massachusetts, United States of America; 7 Department of Otolaryngology and Communication Enhancement, Boston Children’s Hospital, Boston, Massachusetts, United States of America; Institute of Cellular and Organismic Biology, Taiwan

## Abstract

Inner ear sensory hair cells die following exposure to aminoglycoside antibiotics or chemotherapeutics like cisplatin, leading to permanent auditory and/or balance deficits in humans. Zebrafish (*Danio rerio*) are used to study drug-induced sensory hair cell death since their hair cells are similar in structure and function to those found in humans. We developed a cisplatin dose-response curve using a transgenic line of zebrafish that expresses membrane-targeted green fluorescent protein under the control of the *Brn3c* promoter/enhancer. Recently, several small molecule screens have been conducted using zebrafish to identify potential pharmacological agents that could be used to protect sensory hair cells in the presence of ototoxic drugs. Dimethyl sulfoxide (DMSO) is typically used as a solvent for many pharmacological agents in sensory hair cell cytotoxicity assays. Serendipitously, we found that DMSO potentiated the effects of cisplatin and killed more sensory hair cells than treatment with cisplatin alone. Yet, DMSO alone did not kill hair cells. We did not observe the synergistic effects of DMSO with the ototoxic aminoglycoside antibiotic neomycin. Cisplatin treatment with other commonly used organic solvents (i.e. ethanol, methanol, and polyethylene glycol 400) also did not result in increased cell death compared to cisplatin treatment alone. Thus, caution should be exercised when interpreting data generated from small molecule screens since many compounds are dissolved in DMSO.

## Introduction

Sensory hair cells are mechanoreceptors found in the inner ear that detect sound and mediate balance. Loss of sensory hair cells through prolonged noise exposure, aging, and drugs, such as aminoglycoside antibiotics and certain chemotherapeutics, causes permanent hearing deficits in humans. One such chemotherapeutic, cisplatin (cis-diamminedichloroplatinum(II)) is a commonly prescribed platinum-based drug used to treat different types of tumors including testicular, ovarian, cervical, head and neck, and brain cancers [1]. One of the major side effects, however, is irreversible high frequency hearing loss. The overall reported incidence of cisplatin-induced hearing loss is between 28–68% [2] and the variability is due to different risk factors including method of administration (i.e. intravenous), age of the patient, and presence of concurrent treatment with radiotherapy or additional chemotherapeutic agents [1]. Cisplatin ototoxicity in humans is also dose-dependent and cumulative [1].

The zebrafish (*Danio rerio*) is an animal model used to study the signaling pathways regulating sensory hair cell death [3]. Zebrafish have sensory hair cells in inner ear structures that perform both vestibular and auditory functions and a superficial lateral line system that detects vibrations from the surrounding environment [4]. Lateral line hair cells are found in neuromasts along the head, body, and tail and are structurally and functionally similar to mammalian inner ear hair cells. Sensory hair cells in the lateral line neuromasts project into the water, and are readily accessible to pharmacological agents added to the aquatic environment, thereby avoiding drug delivery issues normally associated with *in vivo* model systems. Moreover, several small molecule screens have been used to determine whether certain drugs can ameliorate the effects of different commonly prescribed ototoxic drugs and if they can enhance the regeneration of hair cells in zebrafish neuromasts [3], [5]–[12].

Cells undergoing apoptosis exhibit morphological abnormalities including chromatin condensation, nuclear pyknosis and fragmentation, and plasma membrane blebbing [13]. Cisplatin has been shown to kill zebrafish lateral line hair cells through an apoptotic signaling pathway. Dying hair cells exhibit apoptotic morphological changes [14], [15]. Ou and colleagues (2007) used time-lapse imaging to study cisplatin-induced hair cell death in zebrafish larvae [16] and others have confirmed by ultrastructure analysis that dying zebrafish sensory hair cells exposed to cisplatin undergo apoptosis [15]. Nevertheless, the cellular signaling mechanisms regulating cisplatin-induced hair cell death are still poorly understood [17].

One line of transgenic zebrafish expresses membrane-targeted green fluorescent protein (GFP) under the control of the *Brn3c* promoter/enhancer [10], [18]. The Brn-3 subfamily of POU-domain transcription factor genes consists of 3 homologous members (Brn3a formerly Brn 3.0, Brn3b formerly Brn 3.2, and Brn3c formerly Brn 3.1). In mammals, all three members are expressed in retinal ganglion cells but only Brn3c is expressed in auditory and vestibular hair cells [19], and also in neuromast hair cells in the zebrafish lateral line.

In this study we treated Brn3c-GFP transgenic zebrafish with cisplatin to develop a dose-response curve. Serendipitously, we found that dimethyl sulfoxide (DMSO), a solvent used with many cell death inhibitors (e.g., zVAD) to study aminoglycoside-induced sensory hair cell death in the lateral line [20], potentiated the effects of cisplatin and killed more sensory hair cells than cisplatin alone. DMSO by itself did not kill hair cells. Interestingly, we did not observe synergistic ototoxicity when cisplatin was paired with other organic solvents including methanol, ethanol, or polyethylene glycol 400 (PEG 400), nor when neomycin was paired with DMSO. Finally, we observed more fluorescently-tagged cisplatin in sensory hair cells when the conjugate was solubilized in DMSO rather than with methanol.

## Materials and Methods

### Animals

Wildtype *AB zebrafish (Zebrafish International Resource Center, Eugene, Oregon) and the transgenic TG(Brn3c:GAP43-GFP)^s356t^ fish on the TL background (AKA Brn3c-GFP zebrafish; a gift from Dr. Herwig Baier, University of California San Francisco) [10], [18] were used for these experiments. These zebrafish were maintained on a 14 hour light/10 hour dark cycle and bread using standard procedures in the Harvard University and the Pomona College zebrafish facilities [21]. The embryos were raised in embryo medium until 5 days post-fertilization (dpf) in a 28.5°C incubator (Tritech Research, Los Angeles, CA) [21].

This study was carried out based on recommendations outlined in the Guide for the Care and Use of Laboratory Animals that was issued by the National Institutes of Health. Boston Children’s Hospital Institutional Animal Care and Use Committee (IACUC; Animal assurance number A3303-01) and the Pomona College IACUC (Animal assurance number A3605-01) approved all of the protocols.

For the initial hair cell counts, 5 dpf Brn3c-GFP fish were anesthetized in ethyl 3-aminobenzoate (MS-222; Sigma-Aldrich Corporation, St. Louis, MO) and then fixed in 4% paraformaldehyde (Mallinckrodt Chemical, Hazelwood, MO) at 4°C for 18 to 24 hours. Fixed larvae were rinsed three times in phosphate buffered saline (PBS, Sigma) and immersed in PBS with 0.1% Triton X-100 (PBST; Sigma) for ten minutes. Whole zebrafish larvae were then incubated in Alexa Fluor 594 phalloidin (1∶100; Life Technologies, Carlsbad, CA) for two hours at room temperature, followed by three rinses in PBS, mounted and imaged using a confocal microscope. After it was determined that the GFP tag was sufficient to identify all of the sensory hair cells within the neuromast, zebrafish were no longer co-labeled with phalloidin.

### Cisplatin Treatment

A stock solution of cisplatin (Sigma) was prepared by dissolving solid cisplatin in embryo media. Fresh cisplatin stock solutions were made for each experiment due to its instability in water (Sigma). All solutions were prepared by serial dilution when appropriate. Beginning at 5 dpf, larvae were transferred to 6-well plates (BD Biosciences, San Diego, CA) and incubated in cisplatin for four hours at 0.25 mM, 0.50 mM, 0.75 mM, 1.0 mM, and 1.5 mM [16]. Following drug treatment, we assessed the health of the zebrafish prior to fixation by examining whether each larva had a heartbeat, could swim, and exhibited eye and tail movements. If these parameters were met, then zebrafish were briefly anaesthetized in MS-222 and fixed in 4% paraformaldehyde at 4°C for 18 to 24 hours.

### Detection of Pyknotic Nuclei

After fixation, zebrafish were rinsed three times with PBS for 5 minutes each, permeabilized with PBST for 30 minutes, and then incubated with the cyanine monomeric dye TO-PRO-3 (1∶1000 in PBST; Life Technologies) for two to three hours. TO-PRO-3 binds to DNA and labels nuclei. Zebrafish were rinsed with PBS before being mounted on slides in Vectashield (Vector Laboratories, Burlingame, CA).

### Dimethyl Sulfoxide and Other Organic Solvent Treatments

To determine the concentration at which DMSO has a synergistic effect with cisplatin, DMSO was added to 1 mM cisplatin in embryo medium at concentrations of 0.001%, 0.005%, 0.01%, 0.05%, 0.1%, and 0.5%; 1 mM cisplatin was chosen because this dose effectively kills ∼50% of neuromast hair cells. We chose 0.5% DMSO as our highest concentration since it was the final concentration used with cell death inhibitors during preliminary experiments. Following the four-hour incubation, fish were fixed, processed, and imaged.

Three additional solvents, 0.75% ethanol (Pharmco-AAPER, Brookfield, CT), 0.75% methanol (Sigma) and 0.75% polyethylene glycol 400 (PEG 400; Sigma) were individually added to 1 mM cisplatin to also test for synergistic effects. We chose 0.75% ethanol since doses greater than 0.75% can cause teratogenic effects on the embryo or on sensory hair cells (i.e. fetal alcohol syndrome; data not shown) [22]. After the four-hour incubation, the transgenic larvae were fixed, processed, and imaged.

### Texas Red-cisplatin Treatment

Cisplatin tagged with the fluorophore Texas Red was conceived at Oregon Health & Science University and the synthesis was designed and carried out at Portland State University, adapting the method of Safaei et al. [23] by substituting fluorescein with Texas Red-X succinimidyl ester (mixed isomers; Life Technologies). Preliminary analysis of cisplatin-Texas Red (DDP-TR) uptake in the murine cochlea and cochlear hair cells *in vivo* has been reported elsewhere [24]. Each vial of lyophilized DDP-TR was stored at −20°C and, when needed, dissolved in methanol or DMSO to make a 1 µg/µL stock solution. The DDP-TR stock solution was pipetted vigorously for 2–3 minutes and then sonicated for an additional 5 minutes to completely dissolve the conjugate. The stock solution was diluted with embryo medium to make a final 2 µg/mL working solution. Similarly for the control, unconjugated Texas Red (Life Technologies) was dissolved in either DMSO or methanol to make a 1 µg/µL stock solution and then a 2 µg/mL working solution. In all cases, the final solvent concentration was 0.5%. Embryos were treated with 2 µg/mL DDP-TR or unconjugated Texas Red for 2, 6, 12, 24, or 48 minutes, protected from light exposure, fixed, and co-labeled with TO-PRO-3.

### HPLC and LC/MS Analysis

To determine if DMSO modified the structure of DDP-TR, reversed phase HPLC and LC/MS analysis was performed. Reversed phase HPLC was carried out using a 1525 binary delivery system and a 2996 Photodiode array detector (Waters, MA). LC/MS positive mode analysis was conducted at the Portland State University BioAnalytical Mass Spectrometry Facility, on a ThermoElectron LTQ-Orbitrap Discovery high-resolution mass spectrometer with a dedicated Accela HPLC system.

DDP-TR (0.12 mg) was dissolved in 50 µL of anhydrous methanol. The solution was stirred for four hours at room temperature. Cold diethyl ether (Et_2_O) was added until precipitation occurred. The mixture was centrifuged for five minutes at 4500 rpm, the resulting precipitate was washed with cold Et_2_O (4×2 ml) and dried under vacuum. The resulting precipitate was dissolved in anhydrous methanol and analyzed by reversed phase HPLC and LC/MS positive mode using an analytical Discovery C_18_ column (250×2.1 mm, 5 mm); a solvent gradient water:acetonitrile 95∶5 to 5∶95 in 30 minutes. The wavelength detection was set at 580 nm for the reversed phase HPLC analysis. DDP-TR (0.34 mg) was also dissolved in 100 µL of anhydrous DMSO, and the solution stirred for four hours at room temperature, processed and analyzed by reversed phase HPLC as described above.

### Neomycin Treatment

We treated 5 dpf larvae with DMSO and the aminoglycoside antibiotic neomycin, which kills zebrafish lateral line hair cells [6], [20], [25]–[28]. Five-day post-fertilization larvae were divided into four treatment groups: untreated control, 100 µM neomycin sulfate (Sigma), 0.5% DMSO, and 100 µM neomycin/0.5% DMSO. Additionally, 50 µM neomycin and 50 µM neomycin/0.5% DMSO doses were tested to determine if any synergy was concentration dependent. The dose and duration of neomycin exposure were chosen from a previous study that induced similar hair cell death rates to the cisplatin protocol used in this experiment [25]. In contrast to 1 mM cisplatin treatment for four hours, larvae were treated with neomycin for one hour, washed three times in embryo medium, and allowed to recover in embryo medium for three hours before being fixed with 4% paraformaldehyde, rinsed with PBS, labeled with phalloidin, mounted, and imaged.

### Imaging and Analysis

All zebrafish specimens were whole-mounted in either Vectashield or glycerol and PBS (9∶1) and imaged. Images of lateral line neuromasts were obtained using a Leica TCS SP confocal laser-scanning microscope (Leica Microsystems, Heidelberg, Germany) or a Nikon-C1-SI confocal microscope (Nikon Instruments Inc., Melville, NY). Single images and compressed *z*-series were collected with Leica Software (Leica Microsystems) or EZ-C1 software (Nikon Instruments). Cell counts were performed at the time of imaging by viewing the image slices sequentially. Both the O2 and Mi1 neuromasts were imaged for all of the experiments. These neuromasts were studied since they are easily identifiable and have been used in a previous study [25]. The entire neuromast can be observed in one field of view when the larval zebrafish is mounted on its side. Images were scaled and cropped using Adobe Photoshop (Adobe Systems, San Jose, CA), ImageJ (National Institutes of Health, Bethesda, MD), or EZ-C1 software. Figures were prepared using Adobe Photoshop.

For each set of Texas Red experiments, all specimens in each group of experimental and control tissues were imaged at the same laser intensity and gain settings. Images from each experiment were identically prepared using Adobe Photoshop. Intercellular and extraneous tissue pixels were removed using Photoshop [29]. Fluorescence intensity values in hair cells were obtained from sections by using the pixel histogram function [29].

### Statistics

Quantitative data from hair cell count experiments were subjected to either t-tests for two samples assuming unequal variance (MS Excel, Microsoft Corporation, Redmond, WA) or a one-factorial or two-factorial analysis of variance (ANOVA) using SPSS 12 software (SPSS Inc., Chicago, IL) or VassarStats (Vassar College, Poughkeepsie, NY). Post-hoc comparisons, when appropriate, used the Tukey-Kramer test or Tukey HSD test.

Mann-Whitney nonparametric tests or paired t-tests, depending upon experimental design, were performed between methanol/DDP-TR and DMSO/DDP-TR to identify any statistically significant effect of DMSO on the fluorescent intensity of cellular drug uptake.

## Results

### Brn3c-GFP Transgenic Zebrafish

**Figure 1:**
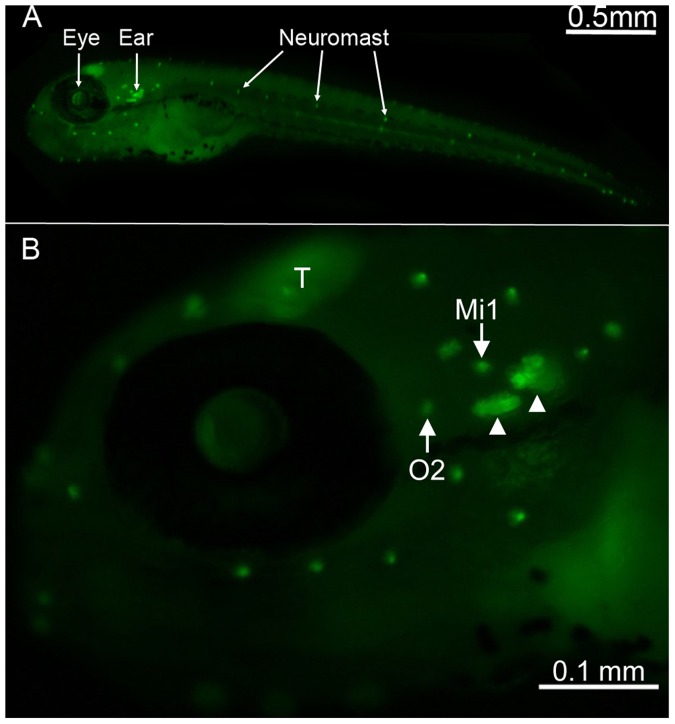
Location of neuromasts on Brn3c-GFP transgenic zebrafish. (A) Lateral view of a 5 days post-fertilization transgenic zebrafish showing GFP expression in neuromasts that are found along the head and the body (bright dots) of the animal. (B) Higher magnification of the head region, with neuromasts containing brightly GFP-labeled hair cells (white arrows) and inner ear organs (white arrowheads) easily identifiable. The otic 2 (O2) and middle 1 (Mi1) neuromasts are highlighted as these are the two neuromasts from which data for this study were obtained. The zebrafish optic tectum (T) is another structure that is also labeled with GFP.

A transgenic zebrafish line expressing membrane-targeted green fluorescent protein (GFP) under the control of the Brn3c promoter/enhancer was used to visualize neuromast hair cells that were clearly distinguishable at low magnification at 5 days post-fertilization (dpf; Fig. 1, arrows). Since zebrafish larvae are relatively transparent, inner ear structures could also be identified using either fluorescence or confocal microscopy (Fig. 1, arrowhead).

Initially, Brn3c-GFP fish were co-labeled with Alexa Fluor 594-conjugated phalloidin (that binds to F-actin abundantly present in stereocilia bundles) to ensure that the GFP-labeled hair cell membranes provided accurate hair cell counts. We counted the number of sensory hair cells in the otic 2 (O2) and middle 1 (MI1) neuromasts [30] since they have been used to investigate aminoglycoside-induced sensory hair cell death [25] and are easily identifiable. The number of phalloidin-labeled hair cells per neuromast was identical to the GFP-labeled neuromasts (n = 22 GFP and phalloidin counts for both O2 and Mi1 neuromasts), similar to previously published data [25]. Thus, using the Brn3c-GFP transgenic zebrafish eliminated the need for phalloidin labeling for F-actin in subsequent experiments.

### Exposure to Cisplatin Causes Dose-dependent Hair Cell Death

**Figure 2:**
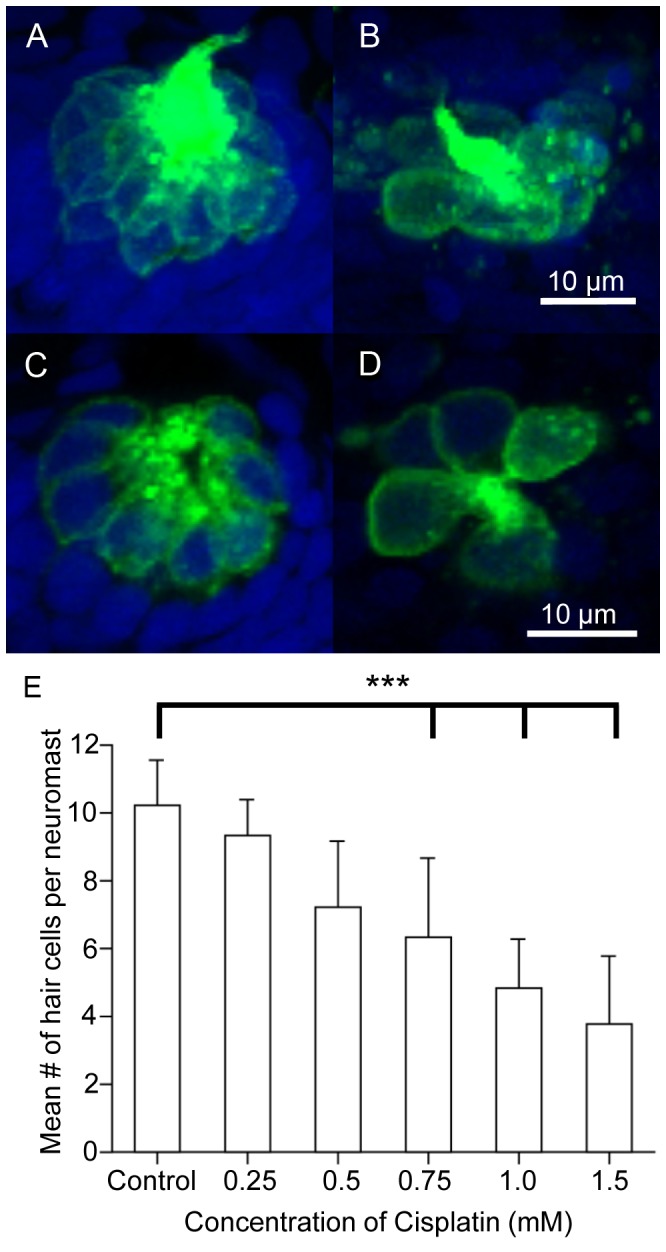
Dose response curve following cisplatin treatment. Five-days post-fertilization Brn3c-GFP transgenic zebrafish were exposed to varying doses of cisplatin for four hours to determine at which dose approximately 50% of the hair cells die. The larvae were fixed, co-labeled with the nuclear dye TO-PRO-3 (blue), and the GFP-tagged hair cells (green) in the O2 neuromast were imaged using confocal microscopy. (A–B) *Z*-stack projections of two O2 neuromasts under different treatment conditions showing the entire neuromast structure. (C–D) Slices from the same neuromasts as in A, B demonstrating the membrane-bound GFP label surrounding the nuclear dye. (A, C) Hair cells appear normal in untreated controls. (B, D) Noticeably fewer hair cells are found in larvae treated with 1 mM cisplatin. (E) The mean number of hair cells per O2 neuromast (± SD) decreased as the dose of cisplatin increased when compared to untreated controls. n = 9−31 neuromasts for each treatment group. ***p<0.001 when individual treatments are compared to untreated controls.

Zebrafish neuromast hair cells are susceptible to cisplatin-induced hair cell cytotoxicity (Fig. 2). Zebrafish larvae were treated with cisplatin (0.25 mM–1.5 mM) for four hours [16], fixed and the surviving GFP-labeled hair cells were imaged and counted. At 5 dpf, control zebrafish contained an average of 10.1±1.7 hair cells in the O2 neuromast. More sensory hair cells were present in untreated controls (Fig. 2A, 2C) than in cisplatin-treated larvae (Fig. 2B, 2C, n = 9−16 for each treatment group). Within four hours, the higher concentrations of cisplatin (0.75 mM, 1 mM, and 1.5 mM) had significantly reduced the number of hair cells in the O2 neuromast (p<0.001). Similar results were obtained for the Mi1 neuromast (data not shown). Therefore, all further experiments were performed using 1 mM cisplatin since that concentration killed roughly 50% of the hair cells in both the O2 and Mi1 neuromasts.

**Figure 3:**
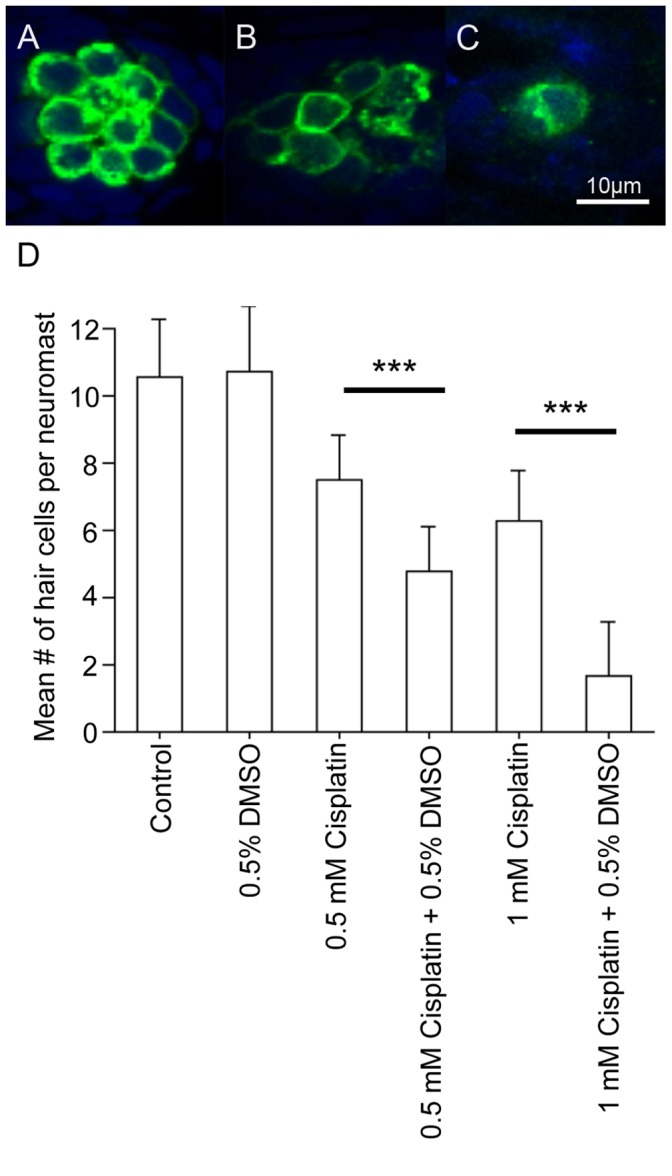
Co-treatment with DMSO and cisplatin compromises hair cell morphology and kills hair cells. Zebrafish were treated with embryo medium (control), 0.5% DMSO, two different concentrations of cisplatin (0.5 mM or 1 mM), or cisplatin (0.5 mM or 1 mM) with 0.5% DMSO. Representative images of (A) control neuromasts, (B) neuromasts exposed to 1 mM cisplatin, and (C) neuromasts exposed to 1 mM cisplatin and 0.5% DMSO. When compared to a control O2 neuromast (A), fewer hair cells were present in the O2 neuromast of a 1 mM cisplatin-treated larva (B). When cisplatin is co-incubated with 0.5% DMSO (C), even fewer hair cells remain within the neuromast. (D) When larvae were treated with 0.5% DMSO in conjunction with cisplatin, there was a significant reduction in the number of hair cells when compared to cisplatin treatment alone indicating a synergistic effect of DMSO and cisplatin. Results are the mean values ± SD. n = 13−51 neuromasts for each treatment group. ***p<0.001.

To further document the toxic effects of cisplatin, the DNA-specific label TO-PRO-3 was used to visualize nuclei (Figs. 2, 3). Pyknotic nuclei appeared smaller and more intense than surrounding nuclei (data not shown). The GFP signal helped determine whether the pyknotic nuclei belonged to hair cells or to the surrounding non-sensory cells. Very few pyknotic nuclei (0.13±0.34) were detected in the O2 neuromast of untreated control larvae. The average number of pyknotic nuclei in GFP-labeled cells increased in a dose-dependent manner when treated with cisplatin. This ranged from 0.55 pyknotic nuclei per neuromast for 0.25 mM cisplatin to 3.27 for 1.5 mM cisplatin (n = 10−16). There was a ten-fold increase in the number of pyknotic nuclei in larvae treated with >0.75 mM cisplatin, which was statistically significant (p<0.05).

### DMSO Increases the Toxicity of Cisplatin

A previous study had shown that a cell death inhibitor was protective against aminoglycoside-induced hair cell death [20]. Interestingly, when cell death inhibitors were used in conjunction with cisplatin, there was increased sensory hair cell death than with cisplatin alone (data not shown). Because cell death inhibitors were dissolved in dimethyl sulfoxide (DMSO), we investigated the possible role of DMSO in cisplatin-induced hair cell death. We chose to use 0.5% DMSO since it was the final concentration used with cell death inhibitors during preliminary experiments.

Hair cells in untreated controls appeared normal (Fig. 3A). Fewer hair cells were present in cisplatin-treated neuromasts (Fig. 3B) but when DMSO was combined with cisplatin, an increase in cell fragmentation and neuromast disorganization was observed (Fig. 3C). Fewer hair cells were present in DMSO/cisplatin-treated larvae, at both 0.5 mM (p<0.001) and 1 mM cisplatin concentrations (p<0.001, n = 13−51 per treatment group). A post-hoc Tukey test revealed 0.5 mM cisplatin plus 0.5% DMSO killed more hair cells than 0.5 mM cisplatin alone, and 1 mM cisplatin plus 0.5% DMSO killed more hair cells than the 1 mM cisplatin alone (Fig. 3C). At the 0.5 mM cisplatin concentration, 5.7±4.8 (DMSO/cisplatin) vs. 8.4±3.0 (cisplatin alone) O2 hair cells remained. At the 1 mM cisplatin concentration, 1.8±1.7 (DMSO/cisplatin) vs. 7.2±2.8 (cisplatin alone) O2 hair cells remained (Fig. 3D). Similar results were obtained for the Mi1 neuromast (data not shown).

**Figure 4:**
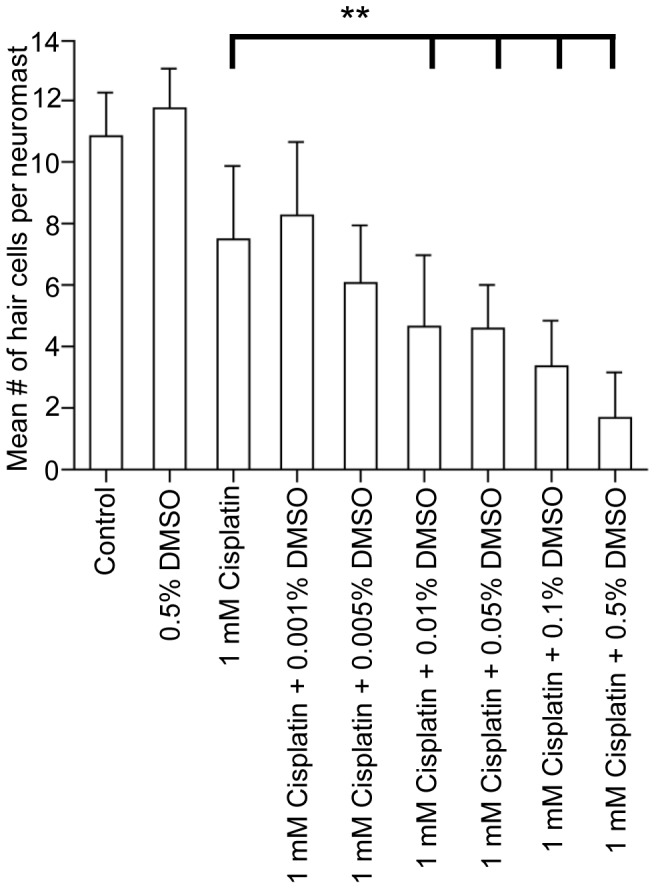
Varying the concentration of DMSO with a constant level of cisplatin affects the number of surviving hair cells present in the neuromast. Brn3c-GFP larvae were treated with either 0.5% DMSO, 1 mM cisplatin or 1 mM cisplatin plus a dose range of DMSO (0.001%–0.5%), fixed, and surviving hair cells in O2 neuromasts were imaged and counted. Increasing the concentration of DMSO reduced the number of O2 sensory hair cells. Larvae treated with 1 mM cisplatin and plus a DMSO dose greater than 0.01% had significantly fewer hair cells than larvae treated with 1 mM cisplatin alone. Results are the mean values ± SD. n = 18−31 neuromasts for each treatment group. **p<0.01 when individual treatments are compared to untreated controls.

When the concentration of DMSO was varied (0.001% to 0.5%) with a concentration of 1 mM cisplatin, we found that the synergistic effect of DMSO appeared at 0.01% (Fig. 4, n = 18−31 per treatment group). DMSO concentrations greater than 0.01% resulted in significantly fewer O2 hair cells when compared to 1 mM cisplatin alone (p<0.05). Similar results were observed in the Mi1 neuromast (data not shown).

When DMSO was used alone, even at higher concentrations (0.25% to 2%), with increased exposure (72 hours), DMSO was not toxic to lateral line hair cells. DMSO by itself did not noticeably change the morphology of the hair cells (data not shown). There were no differences in the number of neuromast hair cells in DMSO-treated larvae compared to untreated controls (data not shown).

### Other Organic Solvents do not Exacerbate the Toxic Effect of Cisplatin

**Figure 5:**
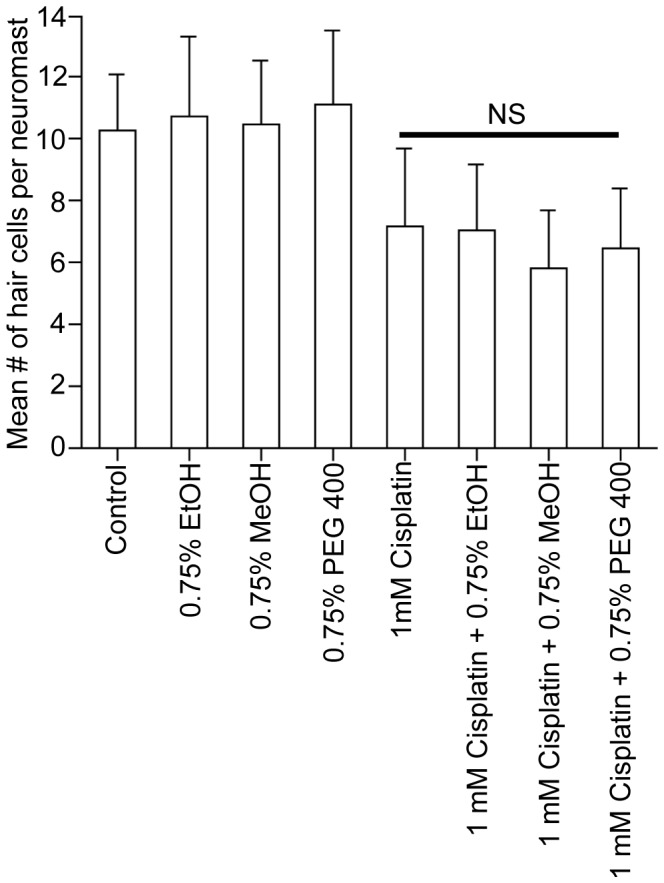
Other organic solvents do not exacerbate the ototoxic effects of cisplatin. Brn3c-GFP zebrafish were treated with 0.75% ethanol (EtOH), 0.75% methanol (MeOH), or 0.75% polyethylene glycol 400 (PEG 400) in the presence or absence of 1 mM cisplatin for four hours. The larvae were then fixed, mounted, and imaged. None of the solvents alone killed hair cells and were similar to control numbers. There was no statistical difference (NS) in the number of surviving hair cells when larvae were treated with 1 mM cisplatin with or without different organic solvents. There was, however, a statistically significant difference (p<0.05) between untreated or solvent-only-treated controls and cisplatin with or without organic solvents. Results are the mean values ± SD. n = 14−42 neuromasts for each treatment group.

We treated 5 dpf larvae with 1 mM cisplatin and either 0.75% ethanol, 0.75% methanol, or 0.75% polyethylene glycol 400 (PEG 400) to determine if other commonly used organic compounds could exacerbate the toxic effect of cisplatin. Concentrations greater than 0.75% for the PEG 400 were lethal to the larval zebrafish during the four-hour incubation period. There was almost no difference in the number of hair cells in the cisplatin-treated larvae and those treated with cisplatin/ethanol, cisplatin/methanol and cisplatin/PEG 400 (Fig. 5, p>0.5, n = 14−42 neuromasts per treatment). Moreover, there were similar numbers of neuromast hair cells in larvae treated only with ethanol, methanol, or PEG 400 as with untreated controls.

### More DDP-TR Fluorescence in Hair Cells Co-treated with DMSO than with Methanol

**Figure 6:**
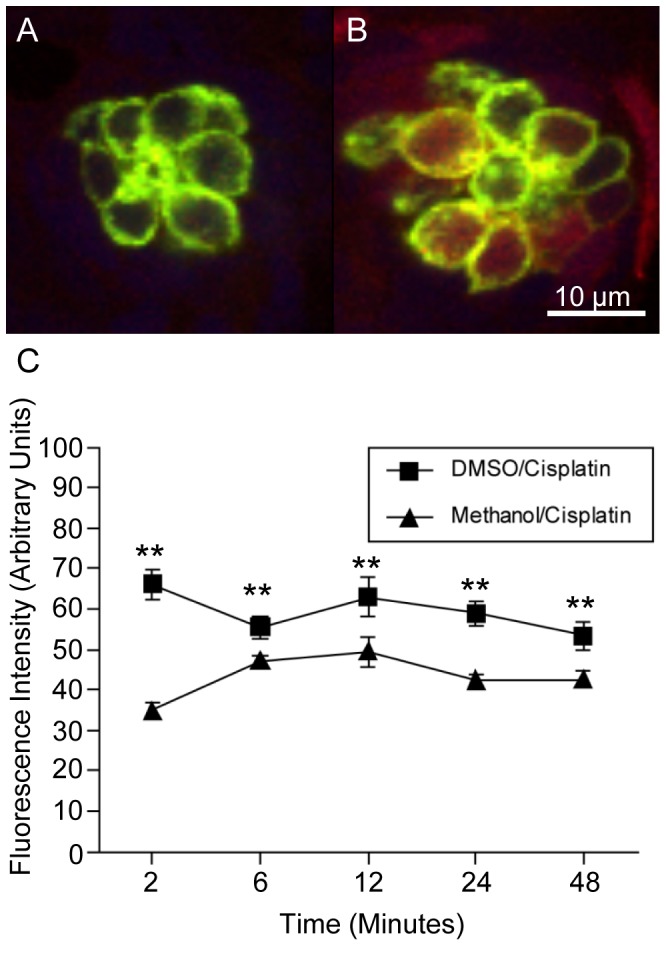
More Texas Red fluorescence in hair cells when DDP-TR is solubilized in DMSO than in methanol. Brn3c-GFP zebrafish were treated with 2 µg/mL DDP-TR dissolved in either methanol or 0.1% DMSO for two to forty-eight minutes and then fixed and imaged. (A) Low levels of DDP-TR fluorescence (red) were found in the O2 hair cells of methanol-treated zebrafish after two minutes. (B) More DDP-TR fluorescence was present in the hair cells of 0.1% DMSO-treated animals after two minutes. (C) DMSO increased levels of DDP-TR fluorescence into neuromast hair cells over time compared to DDP-TR plus methanol-treated fish. Results are the mean values ± SD. n = 15−125 individual hair cells per time point per treatment. **p<0.01.

In order to quantify the levels of cisplatin within lateral line hair cells, we treated the zebrafish with fluorescently tagged cisplatin (DDP-TR; 2 µg/ml) in the presence of either DMSO or methanol for two to forty eight minutes (n = 15−125 individual hair cells per time point per treatment) prior to fixation (Fig. 6). The neuromasts were imaged using identical parameters and fluorescence intensity levels were measured. Methanol/DDP-TR-treated larvae had Texas Red fluorescence (Fig. 6A) in the hair cells, but noticeably more intense Texas Red fluorescence was observed in DMSO/DDP-TR-treated larvae (Fig. 6B). At every time point tested, there was significantly more DDP-TR fluorescence localized in the O2 hair cells of DMSO-treated larvae than in the methanol-treated larvae (p<0.01; Fig. 6C). Similar results were obtained with hair cells in Mi1 neuromasts (data not shown).

As a control experiment, unconjugated Texas Red was used in place of DDP-TR. Zebrafish were treated for 48 minutes with unconjugated Texas Red dissolved with either DMSO or methanol, fixed, and the neuromasts were imaged (data not shown). When using the unconjugated Texas Red, there was no statistical difference (p>0.65) between the fluorescence levels between DMSO-treated zebrafish and methanol-treated zebrafish in hair cells found in both neuromasts (n = 37−47 individual hair cells per time point per treatment). The Texas Red fluorescence levels in these fish were negligible when compared to DDP-TR-treated fish.

**Figure 7:**
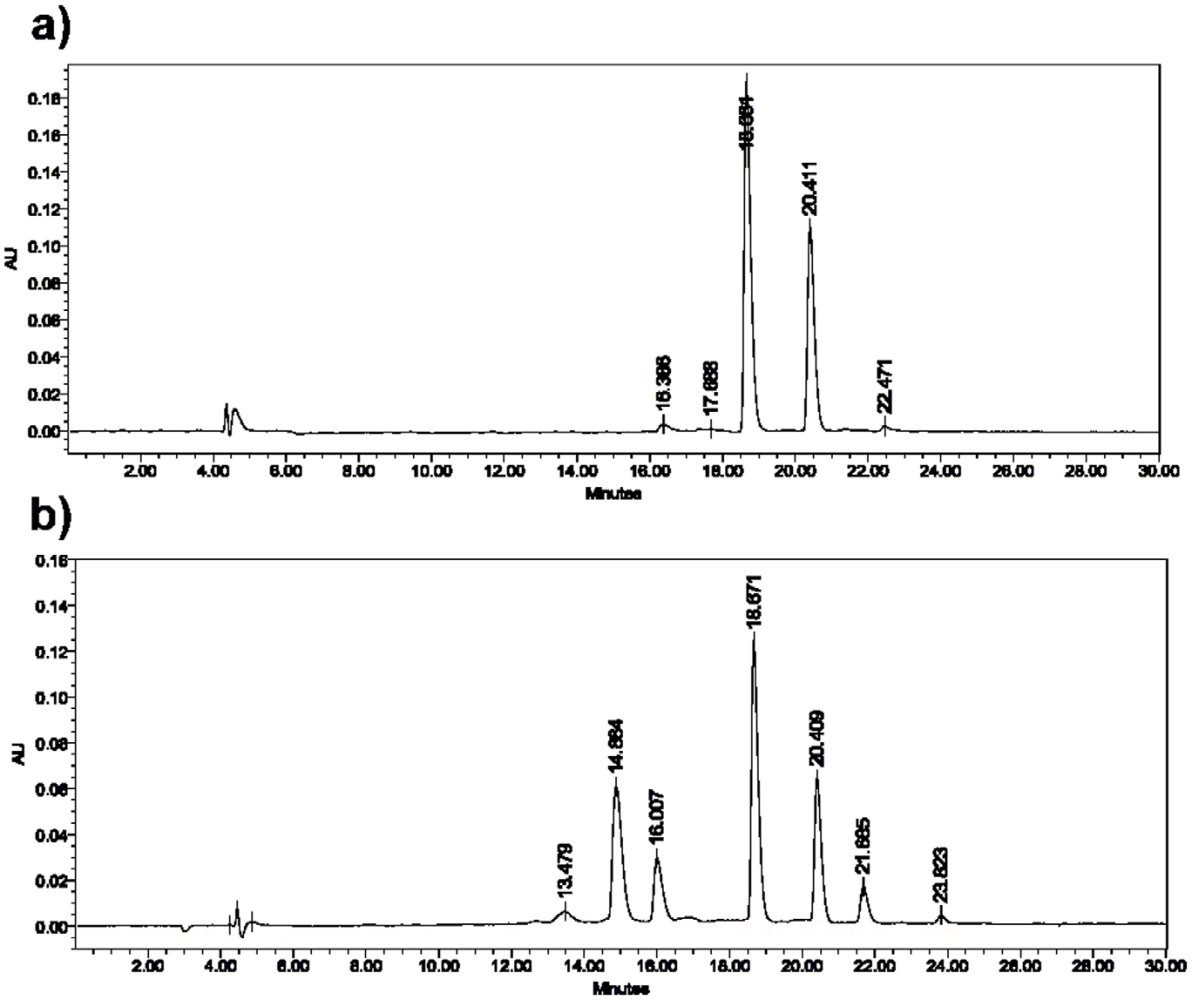
DDP-TR solvated with DMSO has additional elution peaks compared to DDP-TR solvated in methanol. HPLC traces for elution times of DDP-TR-Cl_2_ incubated with (A) methanol or (B) DMSO for four hours at room temperature. Note the two new major peaks at 14.88 and 16.01 minutes in B. HPLC conditions: reversed phase Discovery C_18_ column (250 × 2.1 mm, 5 mm); flow rate: 200 µL, sample volume: 5 µL, water:MeCN. Solvent gradient 95∶5 to 5∶95 in 30 minutes, wavelength detection; 580 nm.

**Figure 8:**
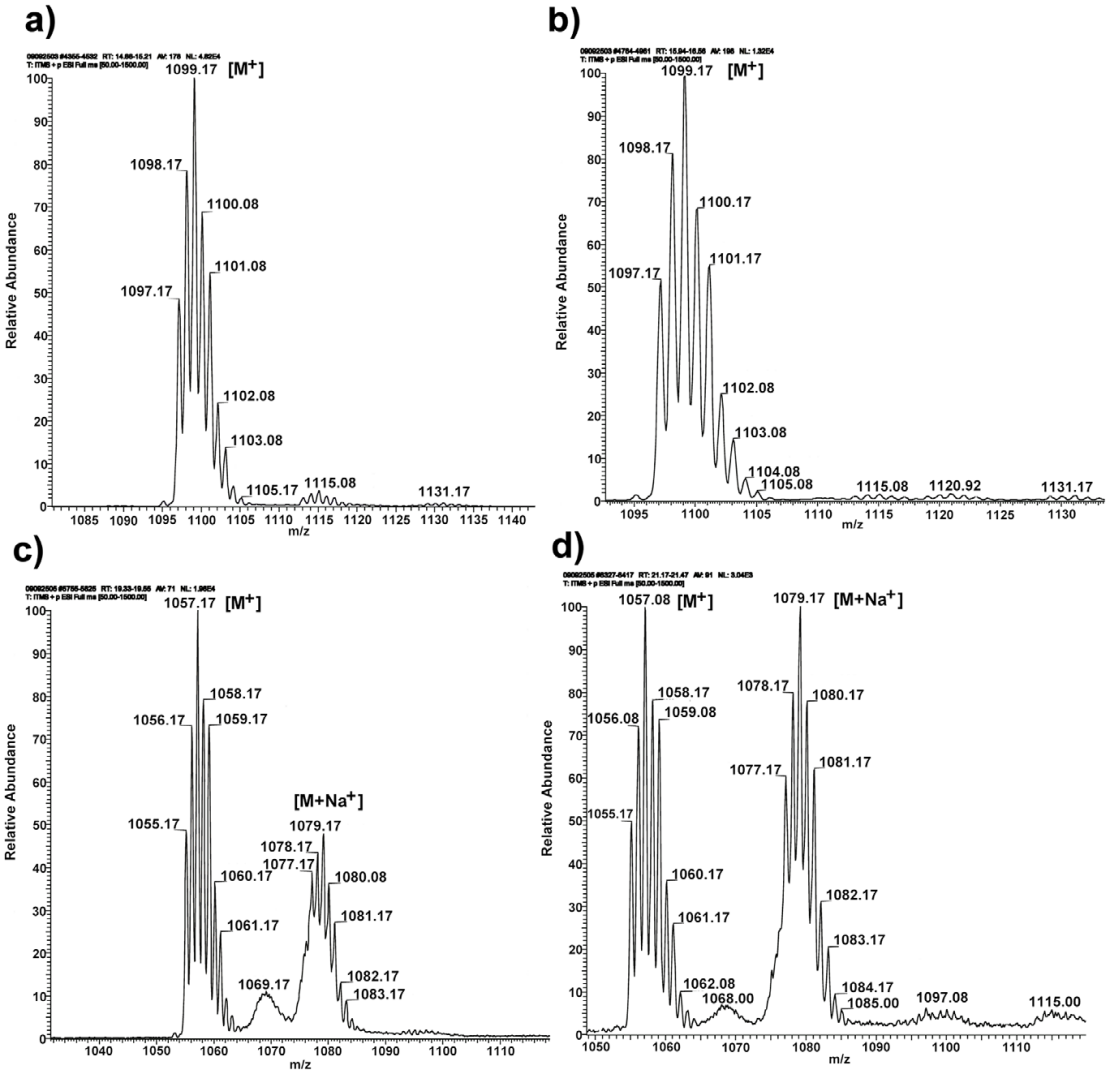
DDP-TR solvated with DMSO has altered spectral peaks compared to DDP-TR solvated in methanol. LR ESI-MS spectra of compounds eluting at (A) 14.88 min corresponding to DDP-TR-Cl-DMSO (i.e., with a DMSO adduct); (B) 16.1 minutes corresponding to DDP-TR-Cl-DMSO; (C) 19.5 minutes corresponding to DDP-TR-Cl_2_, and (D) 21.2 minutes corresponding to DDP-TR-Cl_2_. [M^+^] and [M+Na^+^] ions are observed. M^+^, molecular ion of target compound; M+Na^+^, molecular ion of target compound plus sodium.

Reversed phase HPLC analysis of DDP-TR incubated in methanol for four hours at room temperature demonstrated two isomers of DDP-TR with retention times of 18.7 and 20.4 minutes (Fig. 7A), as expected due to the two isomers of Texas Red present in the conjugation reaction (data not shown). Analysis of DDP-TR after incubation in DMSO for four hours revealed two additional eluting components with retention times of 14.68 and 16.01 minutes (Fig. 7B) in addition to those eluting at 18.7 and 20.4 minutes. Incubation of DDP-TR in DMSO for 18 hours results in almost total conversion of DDP-TR to the components eluting at 14.68 and 16.01 minutes. To determine the molecular identity of the eluting components, LC/LR-ESI-MS in positive mode analysis was conducted. The data in Table 1 indicate that the components eluting at retention times of 19.4 and 22.3 minutes correspond to DDP-TR with the characteristic isotopic pattern of Pt complexes (Fig. 8). Only traces of the DDP-TR-methanol adduct were found. The new components eluting at 14.8 and 16 min observed for DDP-TR incubated in DMSO correspond to the *m/z* of the DDP-TR-DMSO adduct [31], [32]. It is notable that the DDP-TR-DMSO adducts are more polar (with shorter retention/elution times) than DDP-TR.

**Table 1:** Summary of LC/LR-ESI-MS analysis for DDP-TR-Cl_2_ after incubation for 18 hours in methanol (MeOH) and dimethyl sulfoxide (DMSO).

Entry	IncubationSolvent	*Isomer 1*		*Isomer 2*		*m/z*[M^+^]calc.
		*tr*(min)	*m/z*[M^+^]obs.	*tr*(min)	*m/z*[M^+^]obs.	
1	None	19.42	1057.17	21.22	1056.17	DDP-TR; C_40_H_50_Cl_2_N_6_O_7_PtS_2_; 1055.22
2	MeOH	19.45	1057.17	21.23	1057.17	DDP-TR-Cl_2_; C_40_H_50_Cl_2_N_6_O_7_PtS_2_; 1055.22
3	DMSO	14.88	1099.17	16.16	1099.17	DDP-TR-Cl-DMSO; C_42_H_56_ClN_6_O_8_PtS_3_; 1098.26
		19.51	1056.17	21.35	1056.17	DDP-TR-Cl_2_; C_40_H_50_Cl_2_N_6_O_7_PtS_2_; 1055.22

### DMSO does not Increase the Toxicity of the Aminoglycoside Antibiotic Neomycin

**Figure 9:**
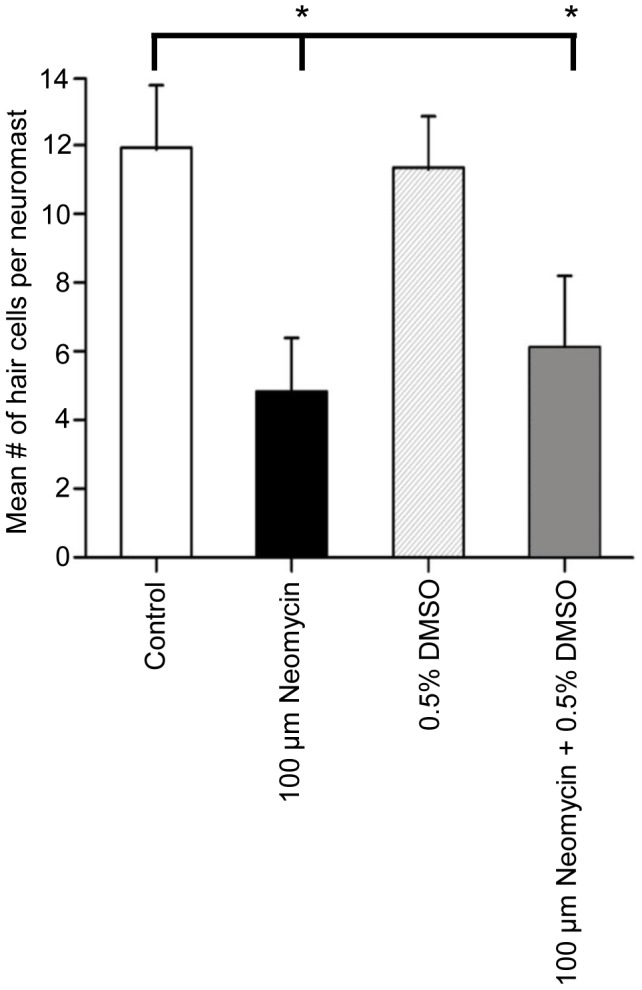
DMSO does not exacerbate neomycin-induced sensory hair cell death. Five dpf zebrafish larvae were treated with embryo medium (white bar), 100 µM neomycin sulfate (black bar), 0.5% DMSO (striped bar), or both for 1 hour (cross-hatched bar), and then washed three times in embryo medium. The larvae were allowed to recover in embryo medium for three hours as described previously [25], and then fixed. Although the neomycin treatment resulted in significantly fewer sensory hair cells, co-treating the larvae with both DMSO and neomycin did not cause more hair cell death than neomycin alone. Results are the mean values ± SD. n = 10−12 neuromasts for each treatment group. *p<0.05 when individual treatments are compared to untreated controls.

To ascertain whether DMSO exacerbated the effects of another commonly used ototoxic drug, we treated 5 dpf larvae with 0.5% DMSO and 100 µM neomycin since many zebrafish studies have used this concentration of neomycin [6], [25]–[28]. Neomycin treatment resulted in a significant decrease in the number of hair cells in both neuromasts of the neomycin and neomycin/DMSO-treated larvae when compared to untreated controls (Fig. 9; p<0.05, n = 10−12 per treatment). Similar numbers of hair cells were found in the neomycin-treated larvae and the neomycin/DMSO-treated larvae (Fig. 9) indicating that DMSO does not act synergistically with neomycin to kill hair cells. Additionally, embryos were exposed to either 50 µM neomycin or 50 µM neomycin/0.5% DMSO to demonstrate that synergy does not exist at a lower dose with no statistically significant difference in the number of hair cells observed (p>0.5, n = 11−14).

## Discussion

Cisplatin killed lateral line hair cells in a dose-dependent manner. Dimethyl sulfoxide (DMSO) combined with cisplatin, however, increased the loss of hair cells beyond that of cisplatin alone; yet DMSO alone did not kill hair cells. Other organic solvents such as methanol, ethanol, and polyethylene glycol 400 did not increase the degree of hair cell death when combined with cisplatin. Finally, DMSO facilitated increased fluorescently-tagged cisplatin (DDP-TR) entry into the hair cells when compared to methanol.

### Cisplatin Toxicity

The dose response curves generated in this study confirm that cisplatin causes hair cell death in a dose-dependant manner. TO-PRO-3 labeling revealed more pyknotic or condensed nuclei in hair cells after cisplatin treatment, consistent with apoptotic-like cell death processes [13]. This supports a previous study, using transmission electron microscopy, that cisplatin induces morphological changes consistent with apoptosis including condensation of the nuclear chromatin in both inner ear and lateral line hair cells of zebrafish [15].

### Synergistic Effect of DMSO

We investigated the role of DMSO in increasing the toxicity of cisplatin. DMSO is a commonly-used solvent for polar and nonpolar compounds (<1,000 daltons), carbohydrates, polymers, peptides, inorganic salts, and gases [33]. DMSO has such excellent solvating power that cisplatin is three orders of magnitude more soluble in DMSO than in water (Sigma).

DMSO could enhance cisplatin’s cytotoxicity by facilitating cisplatin’s entry into hair cells, increasing its intracellular concentration and likelihood of binding to DNA. Little is known about the mechanism of cisplatin uptake by hair cells. Cisplatin entry into hair cells might be increased by an excellent solvent carrier, like DMSO, over endogenous transport alone. Moreover, DMSO reacts with and binds to both cisplatin [31] and DDP-TR. DMSO adducts in cisplatin are formed by substitution with a single chlorine group, forming cis-[Pt(NH_3_)_2_(Me_2_SO)Cl]Cl(1-Cl) and trans-[Pt(NH_3_)_2_(Me_2_SO)Cl]Cl(2-Cl) [31], with a similar reaction for DDP-TR, increasing the polarity of each compound. The greater uptake of DMSO/DDP-TR by neuromast hair cells was only observed at early time points. This is likely due to the limited time exposure to DMSO (and hence adduct formation) prior to treatment of hair cells. Thus, DDP-TR with DMSO-adducts could be taken up faster until depleted, compared to the slower uptake of the relatively more abundant DDP-TR, allowing for the convergence of the fluorescence intensities in the two groups at later time points.

DMSO may also bind to cisplatin after hair cell entry. Cisplatin-DMSO adducts have greater affinity for DNA, potentially increasing its cytotoxicity [31]. DMSO’s effect as a carrier of cisplatin has been studied in various cancer types *in vitro* but the toxic effects of their interaction on hair cells has not yet been shown [33], [34]. Our data show an increased cytotoxicity of cisplatin when using DMSO as a carrier in hair cells of the lateral line. This synergy occurs in a DMSO dose-dependent manner and is unique to DMSO, and was not seen with other solvents. Based on our data showing increased infiltration of DMSO/DDP-TR into the cell, we propose that DMSO facilitates entry of cisplatin into the cell. In contrast, Fischer et al. reported that cisplatin rapidly reacts with DMSO to form a DMSO adduct with reduced DNA binding and reduced cytotoxicity in cancer cells *in vitro* [32]. Decreased tumor toxicity and increased ototoxicity would lessen the practicality of using DMSO for clinical applications.

Contrary to our results, a recent study documented the cytotoxic effect of DMSO, by itself, on hair cells [35]. DMSO caused little or no damage to the hair cells of postnatal rat cochlear organotypic culture that were treated at 0.1% and 0.25% for 24 hours. DMSO concentrations of 0.50% and higher, however, resulted in stereocilia damage, hair cell swelling, and a dose-dependent loss of hair cells. The hair cells exposed to these DMSO concentrations stained positive for TUNEL, caspase-3, caspase-8, and caspase-9, suggestive of apoptotic death. Nevertheless, the authors acknowledged a need for future *in vivo* studies to further assess the possible toxicity of DMSO [35]. The zebrafish *in vivo* experiments reported here provide contrary results to the *in vitro* cochlear organotypic culture study. DMSO treatment alone did not cause hair cell loss or any morphological changes in zebrafish.

The combination of DMSO and neomycin did not cause greater amounts of hair cell death when compared to the group that was treated with neomycin alone. The larvae were not pre-incubated in DMSO as in trials with other inhibitors. While the time course of this experiment differed from the cisplatin experiments, the amount of hair cell death caused by the neomycin was comparable. It is possible that an hour is insufficient for DMSO to facilitate greater levels of neomycin-induced hair cell cytotoxicity.

The experiments presented here lay the groundwork for further studies of cisplatin-induced hair cell death using the Brn3c-GFP transgenic zebrafish. A number of studies have used different candidate therapeutics to inhibit the progression of aminoglycoside-induced hair cell death or accelerate the rate of hair cell regeneration [3], [5]–[8], [10], [11] and one study used the same drugs to screen for cisplatin-induced ototoxicity [9]. Most of these compounds are dissolved in DMSO. Therefore, precautions should be taken if similar screens are used to investigate the signaling mechanisms underlying cisplatin-induced hair cell death since the use of DMSO may increase the likelihood of false negative data. Other studies are needed to determine if these findings involving DMSO and cisplatin are applicable to other animal models.
